# DNA barcoding and morphology reveal a new cryptic species of *Nagiella* (Lepidoptera, Crambidae, Spilomelinae) from Japan

**DOI:** 10.3897/zookeys.1023.60934

**Published:** 2021-03-11

**Authors:** Yuki Matsui, Hideshi Naka, Utsugi Jinbo

**Affiliations:** 1 The United Graduated School of Agricultural Sciences, Tottori University, Tottori, Japan; 2 Faculty of Agriculture, Tottori University, Tottori, Japan; 3 National Museum of Nature and Science, Tsukuba, Ibaraki, Japan

**Keywords:** DNA barcodes, genitalia, *
Patania
*, *
Pleuroptya
*, *Rubus buergeri*

## Abstract

*Nagiella tristalis* Matsui & Naka, **sp. nov.** is described from Japan, based on DNA barcoding and morphological evidence. The two species previously known from Japan, *N. quadrimaculalis* and *N. inferior*, are diagnosed. Photographs of adults, including male and female genitalia of the three species, are provided.

## Introduction

*Nagiella* Munroe, 1976 was established as a replacement name for *Nagia* Walker, 1866 (type species: *Nagia desmialis* Walker, 1866), which is a junior homonym of *Nagia* Walker, 1858 (Lepidoptera, Noctuidae). Munroe (1976) included *Scopula quadrimaculalis* Kollar & Redtenbacher, 1844 and *Sylepta inferior* Hampson, 1899 in this genus, and he described *N. hortulatoides* Munroe, 1976. Munroe also treated the type species, *N. desmialis*, as a synonym of *N. quadrimaculalis*. Ullah et al. (2017) studied this genus in China and described *N. occultalis* Ullah & Yang, 2017, which is a cryptic species of the *N. quadrimaculalis* species complex. Lu and Du (2020) described another species, *N. bispina* Lu & Du, 2020 from China. So far, this genus comprises five species, i.e., *N. quadrimaculalis*, *N. inferior*, *N. hortulatoides*, *N. occultalis*, and *N. bispina*. These species are often confused due to their similar appearance; in these species, the ground color is uniformly greyish and each wing has a conspicuous white spot on each wing, except for *N. hortulatoides*.

Two species, *N. quadrimaculalis* and *N. inferior*, have been recorded in Japan under the genera *Sylepta* Hübner, 1823 (Shibuya 1928, 1929), *Nagia* (Mutuura 1957), and *Pleuroptya* Meyrick, 1890 (Inoue 1982; Sasaki and Yamanaka 2013). In this paper, we describe and illustrate *N. tristalis* sp. nov., a cryptic species of the *N. quadrimaculalis* species complex, from Tottori Prefecture, Japan. We also provide a phylogenetic hypothesis of relationships based on the mitochondrial COI region for the three Japanese *Nagiella* species and *N. occultalis*.

## Material and methods

### Sampling insect specimens

Most specimens of *N. tristalis* were obtained by collecting the larvae in rolled leaves of *Rubus buergeri* Miq. (Rosaceae) during the winter and then rearing them by the method as described below. We also collected the adults of *Nagiella* species and *Patania ruralis* (Scopoli) (to use as the outgroup in the phylogenetic analysis) from various localities of Japan, by light-trap and daytime search. In addition, several specimens of *Nagiella* species were obtained by rearing eggs with the method described below.

Female moths were placed in plastic cups (Clean Cup 129 Pi 860B, with lid Clean Cup 129 Pi FSL [Risupack, Gifu, Japan]; diameter 129 mm, height 130 mm) with fresh leaves of *Rubus buergeri* or *R. trifidus* Thunb. for egg laying. The hatched larvae were reared using fresh leaves of *R. buergeri* or *R. trifidus* under a 14L:10D photoperiod at 25 ± 2 °C and 50–60% relative humidity until pupation, and the resulting pupae were kept in the same conditions until the emergence of adults.

The holotype of the new species is deposited in the National Museum of Nature and Science (NSMT; Tsukuba, Ibaraki, Japan), and the paratypes are stored in the authors’ private collections.

### Genitalia preparation

Before examining the male and female genitalia, the abdomen was detached from the specimen and soaked in a 10% potassium hydroxide (KOH) solution. The soaked abdomen was kept at room temperature overnight and then incubated at 60 °C for 3–6 h. After incubation, the abdomen was transferred into a glass dish with 70% ethanol, and the genitalia were detached from the abdomen under a stereomicroscope (LW-820T; Wraymer Inc., Osaka, Japan) using scissors and tweezers. The genitalia were stained with merbromin in 70% ethanol and mounted on a glass slides in Euparal. The photographs of the whole genitalia were captured with a stereomicroscope (SZX10; Olympus Corp., Tokyo, Japan) and a digital camera (DP25; Olympus Corp., Tokyo, Japan). The magnified views of genital structures were captured by an upright microscope (BX53; Olympus Corp., Tokyo, Japan) with a digital camera (DP21; Olympus Corp., Tokyo, Japan). Genital structures were measured on the screen by Fiji (Schindelin et al. 2012), based on the photographs and whole genitalia lengths measured by a ruler. As references for the terminology, we followed Kristensen (2003) and Kirti and Gill (2007) for the genitalia and Ullah et al. (2017) for the wing maculation.

### DNA extraction, PCR amplification, and sequencing

Total DNA was extracted from the middle legs of the moths using the DNeasy Tissue Kit (Qiagen, Hilden, Germany). The legs were crushed using BioMasher II (FUJIFILM Wako Pure Chemical Co., Osaka, Japan) and incubated with Proteinase K (Takara Bio Corp., Shiga, Japan) for 3–7 d at 60 °C to elute DNA. Subsequent procedures followed the manufacturer’s protocol of the DNeasy Tissue Kit.

The mitochondrial COI gene was amplified using the primers TY-J-1460-Spilo (forward: TACAATTTATCGCTTAATACTCAGCC) and TL2-N-3014-Spilo (reverse: TCCATTACATATAATCTGCCATATTA). These primers were based on TY-J-1460 and TL2-N-3014 (Simon et al. 1994) and were modified for Spilomelinae species based on the whole mitochondrial sequences of *Cnaphalocrocis medinalis* (Guenée, 1854) (accession number: JN246082) and *Haritalodes derogata* (Fabricius, 1775) (accession number: KR233479) from GenBank (https://www.ncbi.nlm.nih.gov/genbank/). The composition of the PCR reaction solution was as follows; 12.5 μl Q5 High-Fidelity DNA Polymerase (New England Biolabs Japan, Tokyo, Japan); 125 nmol of forward and reverse primers; 1 μl DNA extract; and sterilized water was added up to 25 µl in total volume. The PCR amplification was performed in the following programs; initial denaturation phase at 94 °C for 60 s; 40 cycles at 94 °C for 30 s, 54 °C for 60 s, 72 °C 90 s; and final extension phase at 72 °C for 10 min.

The PCR products were checked by electrophoresis on a 1% agarose gel and were purified using NucleoSpin Gel and PCR Clean-up (Takara Bio Corp., Shiga, Japan). Sequencing was conducted at Premix2 analysis service (Fasmac Co., Ltd, Kanagawa, Japan) using the primers LCO1490 (forward: GGTCAACAAATCATAAAGATATTGG) and HCO2198 (reverse: TAAACTTCAGGGTGACCAAAAAATCA) (Folmer et al. 1994). The sequences obtained in this study were deposited into DDBJ (https://www.ddbj.nig.ac.jp/). The accession numbers of these sequences are listed in Table 1.

**Table 1.** Genetic sample information for the material included in this study with accession numbers.

Species	Location	DDBJ accession no.
*Nagiella inferior*	Japan: Yamaguchi, Akiyoshidai	LC527425
*N. inferior*	Japan: Tottori, Wakasa, Hyonosen	LC527427
*N. inferior*	Japan: Shimane, Iinan, Kusandao	LC527428
*N. quadrimaculalis*	Japan: Tottori, Daisen	LC527424
*N. tristalis*	Japan: Tottori, Tottori, Sourokubara	LC527426
*N. tristalis*	Japan: Tottori, Tottori, Uemachi	LC527429
*N. tristalis*	Japan: Tottori, Tottori, Sourokubara	LC527430
*Patania ruralis*	Japan: Tottori, Tottori, Hashimoto	LC527431

### Phylogenetic analysis and BOLD Barcode Index Number clustering

To construct the phylogenetic tree, we downloaded the sequences of *N. inferior*, *N. quadrimaculalis*, and *N. occultalis* (two sequences, respectively) from GenBank. *Patania ruralis* was included as the outgroup because *Patania* (= *Pleuroptya*) is considered to be closely related to *Nagiella* based on male and female genitalia (e.g., Munroe 1976; Inoue 1982), but wing maculation, host plants, and the results of the phylogenetic analysis of Lu and Du (2020) suggest they are clearly different (see also Discussion for the differences between *Patania* and *Nagiella*). The sequences were aligned using MEGA 7.0 (Kumar et al. 2016). A neighbor-joining (NJ) tree was constructed using MEGA 7.0 based on Kimura 2-parameter model (Kimura 1980), and the bootstrap values were calculated with 1,000 replicates. Detection of variation sites and the number of intra/interspecific substitutions were calculated using MEGA 7.0.

DNA barcoding employs DNA sequences in a short and standardized gene region to facilitate species identification. BOLD (http://www.boldsystems.org/) is an international repository of DNA barcodes (Ratnasingham and Hebert 2007). The sequences in BOLD are clustered depending on their divergences and each cluster is given a unique Barcode Index Number (BIN) (Ratnasingham and Hebert 2013), an identifier for DNA barcode-based cluster corresponding to species. We searched the BOLD database for BINs that matched sequences obtained in this study.

## Results

### DNA sequence analysis

We successfully obtained 626 bp sequences of the COI barcode region of the seven specimens of *Nagiella* treated. Variation was detected at 58 sites (9.3%) in these 13 sequences. The number of intraspecific substitutions ranged from 0 to 3 (0–0.5%) while the number of interspecific substitutions ranged from 18 to 41 (2.9–6.5%) (Table 2).

**Table 2.** Mean number of intra (in bold) / interspecific substitutions in mitochondrial COI (626 bp) among four *Nagiella* species.

Species	*Nagiella tristalis*	*N. inferior*	*N. occultalis*	*N. quadrimaculalis*
*Nagiella tristalis* (*n* = 3)	**0.7**			
*N. inferior* (*n* = 5)	35.5	**1.8**		
*N. occultalis* (*n* = 2)	36.7	28.8	**0**	
*N. quadrimaculalis* (*n* = 3)	40.3	36.5	18.3	**0.7**

In the BOLD database, the sequence of *N. quadrimaculalis* obtained in this study corresponds to BOLD:A﻿AD8178, and that of *N. inferior* corresponds to BOLD:AAE4571, while that of *N. tristalis* did not corresponded to any BIN.

**Figure 1.**
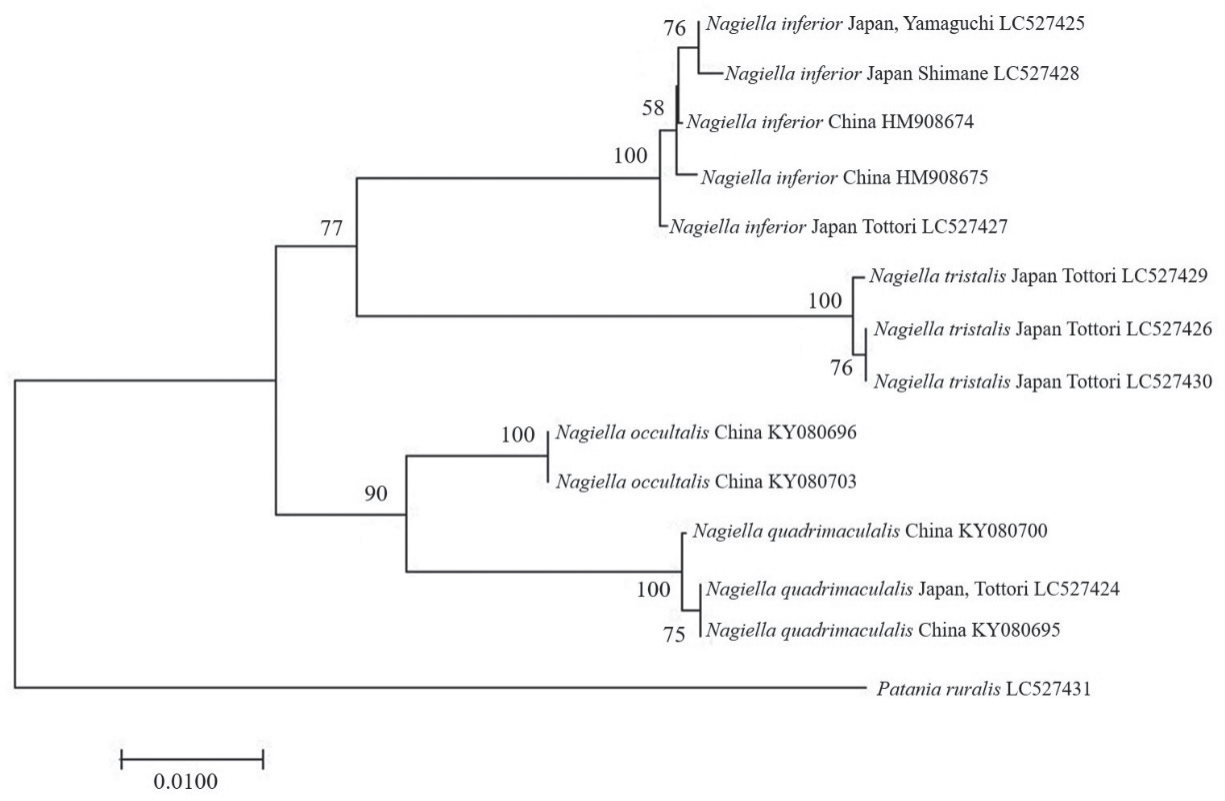
Neighbour-joining (NJ) tree based on mitochondrial COI (626 bp) for 13 sequences of four *Nagiella* species. Nucleotide substitution model based on Kimura 2-parameter model (Kimura, 1980). Bootstrap value was calculated with 1,000 replicates (values <50 are not shown).

The NJ tree (Fig. 1) shows the four monophyletic clades that correspond to morphologically different *Nagiella* species with strong supports (bootstrap value of 100 for each species). The NJ tree also supports the close relationship between *N. tristalis* and *N. inferior* with moderate support (bootstrap value 77), and indicates *N. occultalis* as the sister species of *N. quadrimaculalis* with high support (bootstrap value 90).

### Taxonomy

#### Key to Japanese *Nagiella* species

**Table:** 

1	Forewing length 15.5–18 mm; cilia creamy white at Cu_2_ to A_1+2_ for forewing, Cu_2_ to CuP for hindwing; gnathos of male genitalia slender and elongated; signum of female genitalia with sharp projections at both edges of posterior margin	***N. quadrimaculalis***
–	Forewing length less than 13 mm; cilia of both wings concolorous with ground color; gnathos of male genitalia nearly triangular, short and small; signum of female genitalia small and rounded, without projections	**2**
2	Ground color of both wings lighter, postmedial line distinct especially in the hindwing; large, comma-shaped white spots at end of discal cell in each wing, usually larger; subdiscal white spot of forewing usually quadrilateral, distinct; base of discal cell of hindwing white; valva of male genitalia dorsally straight margined subapically; anterior apophysis of female genitalia slightly incurved to dorsally, expansion of the base sharply triangular; signum of female genitalia circular, small (diameter 0.05–0.06 mm)	***N. inferior***
–	Ground color of both wings darker, postmedial line obscure; large, comma-shaped, white spots at end of discal cell in each wing, usually smaller, especially in the hindwing; subdiscal white spot of forewing rounded, small and blurry; base of discal cell of hindwing concolorous with ground color; dorsal margin of valva of male genitalia slightly incurved subapically; anterior apophysis of female genitalia straight and narrow, expansion of the base broadly triangular; signum of female genitalia nearly elliptic, larger (diameter 0.09–0.14 mm)	***N. tristalis***

#### Nagiella tristalis

Taxon classificationAnimaliaLepidopteraCrambidae

Matsui & Naka sp. nov.

C9D9CE9C-FFB3-503B-98FD-90FDE60D40F4

http://zoobank.org/12764D16E-5465-4859-ADD8-9C1C0C4D06A7

**Figure 2.**
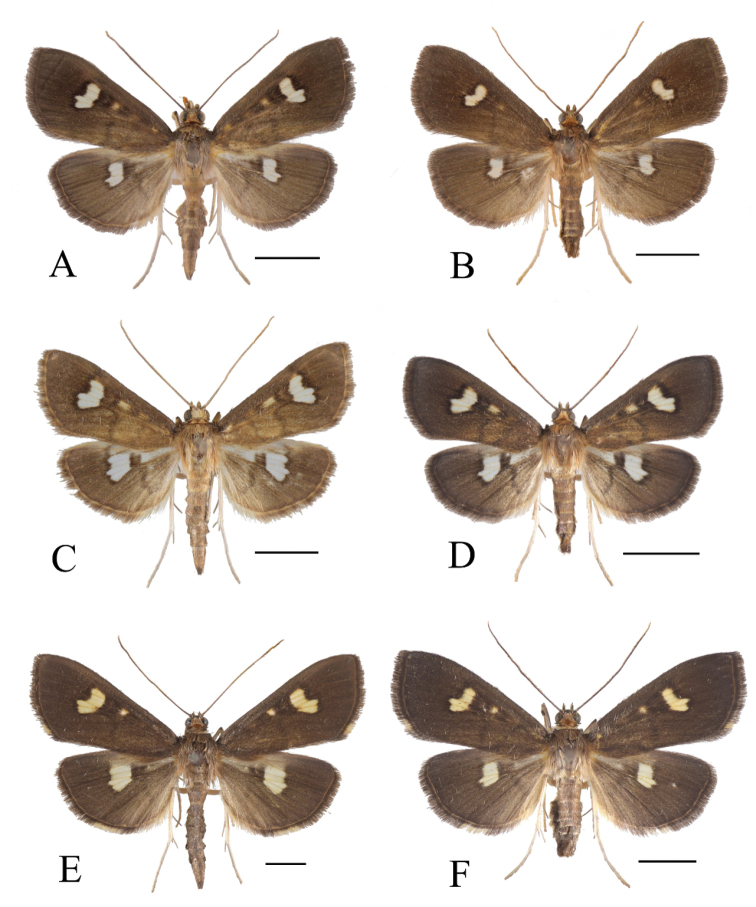
*Nagiella* adults, dorsal view **A***N. tristalis* sp. nov. male **B***N. tristalis* sp. nov. female **C***N. inferior* male **D***N. inferior* female **E***N. quadrimaculalis* male **F***N. quadrimaculalis* female. Scale bars: 5 mm.

**Figure 3.**
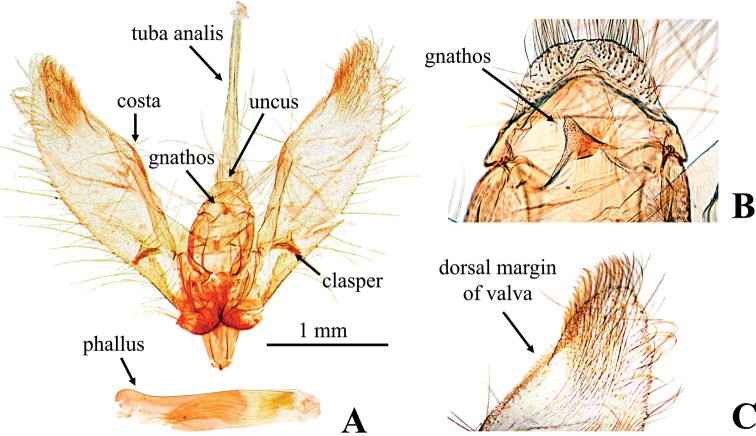
Male genitalia of *Nagiella tristalis* sp. nov. **A** whole genitalia **B** gnathos, magnified view **C** apex of valva, magnified view.

Figures 2A, B , 3A–C , 6A–C

##### Type material.

***Holotype*.** ♂, Japan: Sourokubara, Tottori City, Tottori Pref., 35.46°N, 134.11°E, 110 m, 7 Nov. 2019 (F_1_ emerged), Y. Matsui leg., preserved in National Museum of Nature and Science, NSMT-I-L-75637. ***Paratypes*.** 2♀3♂, Same locality as holotype, 5 Mar. 2018, 6 Apr. 2018, 4 May 2018, 13 Sep. 2018 (emerged), H. Naka, and Y. Matsui leg.; 1♀3♂, Setagura, Tottori City, Tottori Pref., 35.47°N, 134.12°E, 45 m, 7–22 Mar. 2019 (emerged), 23 Sep. 2019 (F_1_ emerged), H. Naka leg.; 2♀, Uemachi, Tottori City, Tottori Pref., 35.50°N, 134.24°E, 40 m, 5 and 10 Feb. 2019 (emerged), Y. Matsui leg.; 1♀, Mt Honjin-yama, Tottori City, Tottori Pref., 35.51°N, 134.26°E, 110 m, 24 Jun. 2012, Y. Matsui leg.; 1♂, Tokumaru, Yazu Town, Tottori Pref., 35.37°N, 134.34°E, 145 m, 20 Aug. 2014, H. Naka leg. ***Other specimens*.** 1♀, Mt Takao, Tokyo To, 19 Jul. 1960, T. Ebato leg. (NSMT-I-L-75536); 1♂, Nashimoto, Shizuoka Pref., 23 May 1953, T. Ebato leg. (NSMT-I-L-75538); 1♂, ditto, 5 Jun. 1959, T. Ebato leg. (NSMT-I-L-75537); 1♂, ditto, 10 Jun. 1961, T. Ebato leg. (NSMT-I-L-75539); 2♂, ditto, 24 Aug. 1966, T. Ebato leg. (NSMT-I-L-75534, 75535); 1♀, Kuragari-Valley, Nukata Town, Aichi Pref., 26 Jun. 1993, A. Sasaki leg. (NSMT-I-L-75593); 1♂, Sugano, Tokuyama City, Yamaguchi Pref., 27 Jun. 1993, T. Ikenoue leg. (NSMT-I-L-75594); 1♀, Shimomyo, Aira Town, Kagoshima Pref., 28 May 1992, Y. Yanagita leg. (NSMT-I-L-75596); 1♀, Kamitsuru, Izumi City, Kagoshima Pref., 14 Jul. 1992, Y. Yanagita leg. (NSMT-I-L-75595); 1♂, Mt Ishizukadake, I. Yakushima, Kagoshima Pref., 5 Aug. 1958, B.T. leg. (NSMT-I-L-75607); 1♂, Nagata, I. Yakushima, Kagoshima Pref., 3 Oct. 2006, M. Owada and T. Fukuda leg. (NSMT-I-L-75606); 1♀, Chuo-rindo, Uken, I. Amamiohshima, Kagoshima Pref., 13 Oct. 1988, M. Owada leg. (NSMT-I-L-75541); 3♂, ditto, 22 Apr. 2009, M. Owada leg. (NSMT-I-L-75609 to 75611); 1♀, Kinsakubaru, Naze, I. Amamiohshima, Kagoshima Pref., 11 Oct. 1988, M. Owada leg. (NSMT-I-L-75542); 1♂, Mt Yuwan-dake, I. Amamiohshima, Kagoshima Pref., 12 Oct. 1988, M. Owada leg. (NSMT-I-L-75543); 1♀, Naze, I. Amamiohshima, Kagoshima Pref., 25 Jun. 1968, Y. Kishida leg. (NSMT-I-L-75540); 1♂, Shinokawa, Setouchi, I. Amamiohshima, Kagoshima Pref., 21 Apr. 2009, M. Owada leg. (NSMT-I-L-75608); 1♂, Mikyo, I. Tokunoshima, Kagoshima Pref., 31 Oct. 1992, M. Owada leg. (NSMT-I-L-75544); 1♂, Gogayama, I. Okinawajima, Okinawa Pref., 30 Mar. 1974, T. Naito leg. (NSMT-I-L-75612); 1♀, Seifuautaki, Chinen-son, I. Okinawajima, Okinawa Pref., 16 Aug. 1980, R. Sato leg. (NSMT-I-L-75613); 1♂, same data as for preceding (NSMT-I-L-75614); 1♀, Ôkuni-bashi, Kunigami-son, I. Okinawajima, Okinawa Pref., 21 Apr. 2001, A. Sasaki leg. (NSMT-I-L-75615).

##### Etymology.

The specific epithet refers to the darker wing color in comparison to that of *N. inferior*, and the habitat of this species is a shaded place.

##### Diagnosis.

This new species is similar to *N. inferior* and *N. quadrimaculalis*, also distributed in Japan, but it can be distinguished by the following characters: length of forewing 12.0–13.0 mm; vertex with erect, dull-orange scales; subdiscal white spot of forewing rounded, small, and blurry; base of discal cell of hindwing identical to ground color; dorsal margin of valva of male genitalia slightly incurved subapically; anterior apophysis of female genitalia straight and narrow; signum of female genitalia nearly elliptical, larger than in *N. inferior* (diameter 0.09–0.14 mm). This species is also similar to *N. occultalis* and *N. bispina* distributed in China, but *N. occultalis* has the following differences: subdiscal white spot of forewing narrowed or elongated, tuba analis of male genitalia sclerotized, gnathos of male genitalia elongated and narrow at the base; *N. bispina* exhibits the following differences: gnathos of male genitalia absent, phallus of male genitalia with a hook-shaped cornutus, corpus bursae of female genitalia with two thorn-like signa. From *N. hortulatoides*, the new species can be easily distinguished by the wing maculation.

##### Description

**(Fig. 2A, B). *Head***: frons brownish grey, smooth. Vertex with erect, dull-orange scales. Labial palpus upturned, dorso-laterally dark brown, ventro-mesally pale white. Antenna dark brown; flagellum filiform with golden cilia ca 1/4 the diameter of flagellum in male.

***Thorax and abdomen*:** dorsally brownish grey; patagium and tegula with ochreous brown. Ventrally milky white.

***Wings*:** length of forewing 12.0–13.0 mm. Ground color of both wings brownish grey, with a large comma-shaped white spot at end of discal cell (the bases of R_5_ to M_3_), that of the hindwing somewhat small; cilia concolorous with ground color; postmedial line obscure. Subdiscal white spot of forewing rounded, small and blurry. Base of discal cell of hindwing concolorous with ground color.

***Male genitalia*** (Fig. 3A–C): uncus short, subtrapezoid, blunt on posterior margin. Gnathos nearly triangular, short and small, apex blunt. Tuba analis elongate, not sclerotized, length ca 0.6 times that of valva. Transtilla subtriangular, completely connected medially. Saccus short, anterior margin rounded. Valva somewhat narrow, length ca 3.3 times that of width, dorsal margin slightly incurved subapically; costa more or less inflated with several setae at apex; clasper large, down curved, the apex blunt. Phallus cylindrical, without cornutus.

***Female genitalia*** (Fig. 6A–C): ovipositor lobe oblong, setose. Anterior apophysis ca 2 times length of posterior apophysis, narrow, almost straight, with triangular expansion near base. Antrum trapezoid, sclerotized at collar. Ductus bursae almost equal in length to corpus bursae, membranous with wrinkles, slightly narrowed at posterior end. Corpus bursae pear-shaped, membranous; signum nearly elliptical, sparsely covered with denticles.

##### Biology.

In Honshu, Japan, adults are found in May to September, and they are considered bivoltine. They appear to be hardly attracted to light. Larvae feed on *Rubus buergeri* and the middle instar larvae overwinter in its leaves.

##### Distribution.

Japan: Honshu (Tokyo, Shizuoka, Aichi, Tottori, Yamaguchi), Kyushu (Kagoshima), Ryukyu Islands (Yakushima, Amamioshima, Tokunoshima, Okinawajima).

##### Remarks.

The shapes of the uncus and gnathos show intraspecific variations, i.e., in several specimens, the posterior margin of the uncus is slightly notched medially, and the projection of the gnathos is smaller than that shown on Figure 3B. Also, in *N. inferior*, the shape of the gnathos varies similarly as in *N. tristalis*. Therefore, these characters are unsuitable for diagnosis of *N. tristalis* and *N. inferior*. Similarly, Ullah et al. (2017) proposed the shape of the claspers as a diagnostic character for distinguishing between *N. occultalis* and *N. quadrimaculalis*, but we could not find any clear difference among *N. tristalis*, *N. inferior*, and *N. quadrimaculalis*, because of the overlap in intraspecific variations.

#### Nagiella inferior

Taxon classificationAnimaliaLepidopteraCrambidae

(Hampson, 1899)

F362FB0F-F6C4-5C70-B370-02DBB5C72348

**Figure 4.**
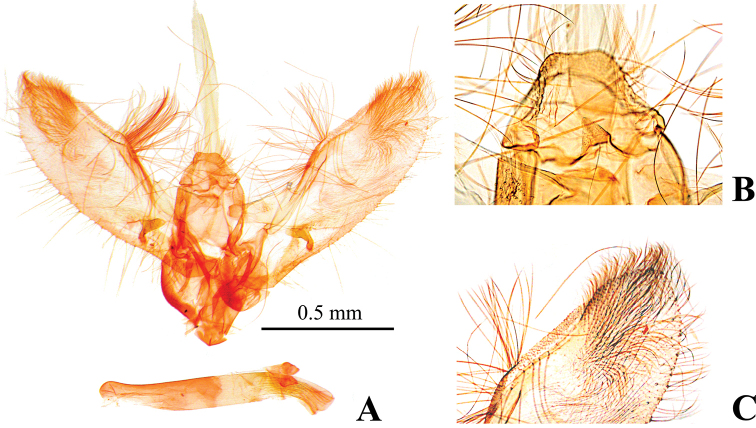
Male genitalia of *Nagiella inferior***A** whole genitalia **B** gnathos, magnified view **C** apex of valva, magnified view.

Figures 2C, D , 4A–C , 7A–C

**Figure 5.**
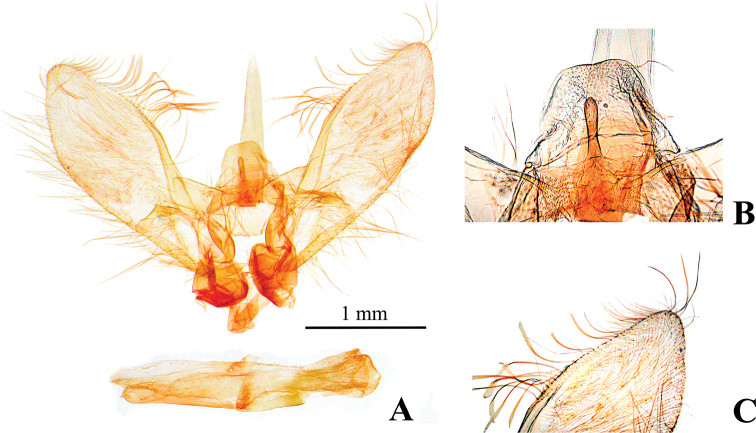
Male genitalia of *Nagiella quadrimaculalis***A** whole genitalia **B** gnathos, magnified view **C** apex of valva, magnified view.

Botys quadrimaculalis Motschulsky, 1861: 1: 37 (preoccupied).
Pleuroptya quadrimaculalis : Bae, 2001: 122–124, pl. 5 fig. 172; Park et al. 2007: 177, 239 fig. 94; Bae et al. 2008: 148–149, fig. 167.
Sylepta inferior Hampson, 1899: 724; Shibuya 1928: 228; Shibuya 1929: 189.
Nagia inferior : Mutuura, 1957: 122, pl. 21 fig. 636.
Nagiella inferior : Munroe, 1976: 878; Lu and Du 2020: 149, fig. 5, figs 5, 10, 13.
Pleuroptya inferior : Inoue, 1982: 1: 343; 2: 234, 454, pl. 40, fig. 16; Li et al. 2012: 625–626, pl. 18, fig. 416; Sasaki and Yamanaka 2013: 81, 451.

##### Material examined.

Japan: 1♂, Tohro, Hokkaido, 3 Jul. 1962, T. Ebato leg., (NSMT-I-L-75498); 1♂, Riv. Rusagawa, Shiretoko, Hokkaido, 26 Jul. 1962, K. Tsuchiya leg. (NSMT-I-L-75523); 1♂, Sapporo, Hokkaido, 6 Jul. 1933, collector unknown (NSMT-I-L-75529); 1♂, Shumarinai, Hokkaido, 20 Jul. 1998, Y. Kishida leg. (NSMT-I-L-75569); 1♂, Shibecha, Hokkaido, 8 Jul. 1958, K. Jinbo leg. (NSMT-I-L-75574); 2♂, Akan, Hokkaido, 13 Jul. 1958, K. Jinbo leg. (NSMT-I-L-75575, 75576); 1♂, Toubai, Nemuro City, Hokkaido, 5 Aug. 2013, U. Jinbo leg. (NSMT-I-L-37577); 1♂, Sannai-Ishizawa, Honjoh City, Akita Pref., 29 Jun. 1975, A. Sasaki leg. (NSMT-I-L-75548); 1♂, ditto, 29 Jun. 1978, A. Sasaki leg. (NSMT-I-L-75545); 2♂, ditto, 15 Jun. 1979, A. Sasaki leg. (NSMT-I-L-75546, 75547); 1♀, Shinzan Park, Honjoh City, Akita Pref., 11 Jul. 1977, A. Sasaki leg. (NSMT-I-L-75549); 1♂, Uwanodai, Kawabe Town, Akita Pref., 5 Jul. 1977, A. Sasaki leg. (NSMT-I-L-75555); 1♀, ditto, 12 Jul. 1977, A. Sasaki leg. (NSMT-I-L-75551); 1♀, ditto, 26 Jun. 1978, A. Sasaki leg. (NSMT-I-L-75552); 1♀, ditto, 19 Jul. 1979, A. Sasaki leg.; 1♂, ditto, 19 Jul. 1979, A. Sasaki leg. (NSMT-I-L-75556); 1♂, ditto, 11 Jun. 1980, A. Sasaki leg. (NSMT-I-L-75550); 1♂, ditto, Akita Pref., 2 Jul. 1980, A. Sasaki leg. (NSMT-I-L-75553); 1♀, Kamibiguchi, Gojohme Town, Akita Pref., 26 Jul. 1979, A. Sasaki leg. (NSMT-I-L-75557); 1♂, Tazawako Height, Tazawako Town, Akita Pref., 20 Aug. 1979, A. Sasaki leg. (NSMT-I-L-75558); 1♂, Chikogi-zaki, Hachimori Town, Akita Pref., 13 Jul. 1979, A. Sasaki leg. (NSMT-I-L-75559); 1♀, Niida, Akita City, Akita Pref., 16 Jun. 1978, A. Sasaki leg. (NSMT-I-L-75560); 1♂, Asahimata, Akita City, Akita Pref., 16 Jul. 1980, A. Sasaki leg. (NSMT-I-L-75561); 1♂, Nibetsu, Akita City, Akita Pref., 3 Jul. 1980, A. Sasaki leg. (NSMT-I-L-75562); 1♂, ditto, 23 Jun. 1987, A. Sasaki leg. (NSMT-I-L-75563); 1♂, Mt Takao, Yuma Town, Akita Pref., 8 Jul. 1985, A. Sasaki leg. (NSMT-I-L-75564); 1♂, Tōshi, Nikaho Town, Akita Pref., 18 Jul. 1995, A. Sasaki leg. (NSMT-I-L-75565); 1♂, ditto, 19 Aug. 1984, A. Sasaki leg. (NSMT-I-L-75566); 1♂, Matsusaka, Ōno-dai, Moriyoshi Town, Akita Pref., 13 Jul. 1996, A. Sasaki leg. (NSMT-I-L-75567); 1♀, Aburato, Tsuruoka City, Yamagata Pref., 11 Jul. 1990, A. Sasaki leg. (NSMT-I-L-75568); 1♂, Futamata-Spa, Fukushima Pref., 6 Aug. 1967, T. Ebato leg. (NSMT-I-L-75497); 1♂, Shiozawa-Spa, Fukushima Pref., 5 Aug. 1967, T. Ebato leg. (NSMT-I-L-75501); 1♂, ditto, 6 Aug. 1967, T. Ebato leg. (NSMT-I-L-75519); 1♂, ditto, 29 Jun. 1968, T. Ebato leg. (NSMT-I-L-75518); 2♂, Hanashiki-Spa, Gunma Pref., 22 Jun. 1963, T. Ebato leg. (NSMT-I-L-75459, 75460); 1♀, same data as for preceding, (NSMT-I-L-75487); 1♂, ditto, 8 Jul. 1961, T. Ebato leg. (NSMT-I-L-75492); 3♂, Uenohara, Gunma Pref., 8 Jul. 1961, T. Ebato leg. (NSMT-I-L-75488 to 75490); 1♂, Kawaburu-Spa, Gunma Pref., 1 Jul. 1967, T. Ebato leg. (NSMT-I-L-75491); 1♂, Kitakaruizawa, Gunma Pref., 12 Jul. 1970, T. Okada leg. (NSMT-I-L-75528); 1♂, Minakami, Gunma Pref., 22 Jul. 1931, collector unknown (NSMT-I-L-75530); 1♀, same data as for preceding (NSMT-I-L-75531); 1♂, Mt Mitsumine, Saitama Pref., 15 Jul. 1961, T. Ebato leg. (NSMT-I-L-75461); 1♀, Shikanoyu, Titibu, Saitama Pref., 26 Jul. 1933, collector unknown (NSMT-I-L-75532); 1♂, Bushi, Iruma City, Saitama Pref., 10 Sep. 1979, H. Inoue leg. (NSMT-I-L-75635); 1♂, Kameyama, Chiba Pref., 12 Aug. 1963, T. Ebato leg. (NSMT-I-L-75517); 1♂, Kiyose, Tokyo To, 10 Jun. 1956, T. Ebato leg. (NSMT-I-L-75502); 2♀, ditto, 18 Aug. 1958, T. Ebato leg. (NSMT-I-L-75503, 75504); 1♀, ditto, 15 Aug. 1959, T. Ebato leg. (NSMT-I-L-75505); 1♀, ditto, 2 Jul. 1959, T. Ebato leg. (NSMT-I-L-75506); 1♂, ditto, 12 Jun. 1958, T. Ebato leg. (NSMT-I-L-75507); 1♀, ditto, 31 Aug. 1958, T. Ebato leg. (NSMT-I-L-75508); 1♀, ditto, 18 Aug. 1959, T. Ebato leg. (NSMT-I-L-75509); 1♀, ditto, 2 Jul. 1957, T. Ebato leg. (NSMT-I-L-75511); 1♂, ditto, 4 Jun. 1958, T. Ebato leg. (NSMT-I-L-75512); 1♀, Ohizumi, Tokyo To, 18 Aug. 1966, T. Ebato leg. (NSMT-I-L-75513); 2♂, Mt Takao, Tokyo To, 27 Jun. 1959, T. Ebato leg. (NSMT-I-L-75514, 75515); 1♂, ditto, 7 Jun. 1961, T. Maenami leg. (NSMT-I-L-75527); 1♂, ditto, 19 Jun. 1996, U. Jinbo leg. (NSMT-I-L-33621); 1♂, Mt Mihara, Tokyo To, 31 May 1962, R. Aoki leg. (NSMT-I-L-75521); 1♀, Mt Mitake, Tokyo To, 16 Jul. 1960, T. Maenami leg. (NSMT-I-L-75526); 1♂, ditto, 20 Aug. 1959, T. Ebato leg. (NSMT-I-L-75510); 1♀, ditto, 25 Jul. 1998, U. Jinbo leg. (NSMT-I-L-36058); 1♂, Institute of Nature Study, Minato-ku, Tokyo To, 6 Jun. 2017, U. Jinbo leg. (NSMT-I-L-55639); 1♂, Hodokubo, Hino City, Tokyo To, 3 Jun. 1990, U. Jinbo leg. (NSMT-I-L-75626); 1♀, ditto, 16 Jun. 1990, U. Jinbo leg. (NSMT-I-L-75627); 1♂, ditto, 3 Aug. 1991, U. Jinbo leg. (NSMT-I-L-75628); 1♀, ditto, 7 Sep. 1991, U. Jinbo leg. (NSMT-I-L-75629); 1♀, ditto, 23 Aug. 1992, U. Jinbo leg. (NSMT-I-L-75630); 1♂, Yokozawairi, Itsukaichi Town, Tokyo To, 18 Jun. 1994, U. Jinbo leg. (NSMT-I-L-75631); 1♂, ditto, Tokyo To, 27 Aug. 1994, U. Jinbo leg. (NSMT-I-L-75632); 1♀, Imperial Palace, Chiyoda-ku, Tokyo To, 26 May 2009, Y. Arita et al. leg. (NSMT-I-L-22523); 1♂, ditto, 7 Sep. 2010, [Malaise trap] (NSMT-I-L-22525); 1♂, ditto, 9 Aug. 2011, [Malaise trap] (NSMT-I-L-28051); 1♀, ditto, 4 Sep. 2012, Y. Arita, H. Nakajima, M. Owada, Y. Kishida and U. Jinbo leg. (NSMT-I-L-31522); 1♂, Noborito, Tokyo To, 27 May 1932, collector unknown (NSMT-I-L-75533); 1♂, Nishitanzawa, Kanagawa Pref., 18 Jun. 1966, Y. Kishida leg. (NSMT-I-L-75525); 1♀, Mt Myôjô-san, Itoigawa City, Niigata Pref., 7 Aug. 1999, A. Sasaki leg. (NSMT-I-L-75570); 1♂, Teradomari, Niigata Pref., 9 Aug. 2005, R. Sato leg. (NSMT-I-L-75571); 1♂, ditto, 7 Jun. 2005, T. Naito leg. (NSMT-I-L-75572); 1♂, Yuzurihara, Yamanashi Pref., 23 Jun. 1945, T. Ebato leg. (NSMT-I-L-75499); 1♂, Kiyosato, Yamanashi Pref., 22 Jul. 1967, T. Ebato leg. (NSMT-I-L-75500); 1♂, Karuizawa, Nagano Pref., 21 Jul. 1958, T. Ebato leg. (NSMT-I-L-75493); 1♂, Miyota, Nagano Pref., 17 Aug. 1965, T. Ebato leg. (NSMT-I-L-75494); 1♂, Tobira-Spa, Nagano Pref., 14 Jul. 1957, T. Ebato leg. (NSMT-I-L-75495); 1♀, Kumanotaira, Nagano Pref., 7 Jul. 1962, T. Ebato leg. (NSMT-I-L-75496); 1♂, ditto, 25 Jun. 1944, H. Inoue leg. (NSMT-I-L-75520); 1♂, Nashimoto, Shizuoka Pref., 10 Jun. 1961, T. Ebato leg. (NSMT-I-L-75516); 2♂, Asagiri Plateau, Shizuoka Pref., 35.42°N, 138.59°E, 910 m, 27 Aug. 2019, Y. Matsui leg.; 1♂, Gujo-Rokunori, Gifu Pref., 5 Jul. 1966, S. Sawatani leg. (NSMT-I-L-75573); 1♂, Mt Hyôno-sen, Wakasa Town, Tottori Pref., 35.35°N, 134.49°E, 860 m, 6 Nov. 2017 (F_1_ emerged), Y. Matsui leg.; 1♂, ditto, 21 Jun. 2020, Y. Matsui leg.; 1♂, Hirodomeno, Wakasa Town, Tottori Pref., 35.41°N, 134.45°E, 800 m, 4 Sep. 2015, Y. Matsui leg.; 4♀6♂, Tokumaru, Yazu Town, Tottori Pref., 35.37°N, 134.34°E, 145 m, 9–11 Aug. 2013, 10–20 Oct. 2014 (F_1_ emerged), H. Naka leg.; 2♀, Wakabadai-kita,Tottori City, Tottori Pref., 35.45°N, 134.26°E, 40 m, 6 Jun. 2012, 22 Aug. 2014, Y. Matsui leg.; 1♂, Kôchi, Shikano Town, Tottori City, Tottori Pref., 35.40°N, 134.00°E, 495 m, 12 Jun. 2020, Y. Matsui leg.; 3♂, Mt Daisen, Kôfu Town, Tottori Pref., 35.35°N, 133.55°E, 910 m, 30 Sep.–2 Oct. 2017 (F_1_ emerged), Y. Matsui leg.; 2♂, Mt Senjô-san, Kotoura Town, Tottori City, 35.43°N, 133.60°E, 385 m, 15 Jul. 2019, Y. Matsui leg.; 1♂, Ichibata, Izumo City, Shimane Pref., 27 May 1967, T. Maenami leg. (NSMT-I-L-75522); 1♀, same data as for preceding (NSMT-I-L-75524); 1♀1♂, Kusandao, Înan Town, Shimane Pref., 35.06°N, 132.83°E, 915 m, Oct. 2017 (F_1_ emerged), Y. Matsui leg.; 1♀, Sugano, Tokuyama City, Yamaguchi Pref., 3 Jun. 1994, T. Ikenoue leg. (NSMT-I-L-75577); 1♀, Yunoki, Tokuji Town, Yamaguchi Pref., 15 Jul. 1995, T. Ikenoue leg. (NSMT-I-L-75578); 2♀, Mt Tokusagamine, Yamaguchi Pref., 3 Aug. 1996, T. Ikenoue leg. (NSMT-I-L-75579, 75580); 1♂, Jakuchikyô, Yamaguchi Pref., 27 Jul. 1995, T. Ikenoue leg. (NSMT-I-L-75581); 1♂, Akiyoshi-dai, Mine City, Yamaguchi, 34.24°N, 131.31°E, 240 m, 16 Sep. 2018, Y. Matsui leg.; 1♀, Kamitsuru, Izumi City, Kagoshima Pref., 14 Jul. 1992, Y. Yanagita leg. (NSMT-I-L-75582); 1♀, Shin-Wase-Tunnel, I. Amamiohshima, Kagoshima Pref., 27 Mar. 2009, M. Owada and M. Kimura leg. (NSMT-I-L-75617); 1♂, Yona, I. Okinawajima, Okinawa Pref., 1 Apr. 1964, T. Nagano leg. (NSMT-I-L-75618); 1♂, Seifuautaki, Chinen-son, I. Okinawajima, Okinawa Pref., 8 Aug. 1980, R. Sato leg. (NSMT-I-L-75619); 2♂, Mt Terukubi-yama, Kunigami-son, I. Okinawajima, Okinawa Pref., 10 Aug. 1980, R. Sato leg. (NSMT-I-L-75620, 75621); 1♂, Haneji, Nago City, I. Okinawajima, Okinawa Pref., 17 Aug. 2001, M. Kimura leg. (NSMT-I-L-75622); 1♂, ditto, 9 Aug. 2002, M. Kimura leg. (NSMT-I-L-75623); 1♂, ditto, 12 Aug. 2002, M. Kimura leg. (NSMT-I-L-75624); 1♂, Takeda-Rindo, I. Ishigakijima, Okinawa Pref., 5 Jun. 2007, M. Kimura leg. (NSMT-I-L-75625).

##### Diagnosis.

Adult (Fig. 2C, D). Forewing length 9.0–12.5 mm. This species is similar to *N. tristalis* and *N. quadrimaculalis*, but can be distinguished by the following characters: forewing shorter; vertex scales lighter than in *N. tristalis* and *N. quadrimaculalis*; subdiscal white spot of forewing usually quadrilateral and distinct; base of discal cell of hindwing broadly white; gnathos of male genitalia nearly triangular, short and small, apex rounded (Fig. 4B); valva of male genitalia dorsally straight margined subapically (Fig. 4C); anterior apophysis of female genitalia slightly curved to dorsally, expansion of the base is sharply triangular (Fig. 7B); signum of female genitalia circular, smaller than in *N. tristalis* (diameter 0.05–0.06 mm) (Fig. 7C). This species is also similar to *N. occultalis*, but *N. occultalis* has the following differences: forewing length 15–16 mm; subdiscal white spot of forewing narrowed or elongated; base of discal cell of hindwing concolorous with ground color; tuba analis of male genitalia sclerotized; gnathos of male genitalia elongated and narrow at the base. Lu and Du (2020) also mentioned genital differences between these species, and as far as we can see from the specimen image by Lu and Du (2020), *N. bispina* is externally distinguishable from this species by base of discal cell of the hindwing being concolorous with the ground color.

##### Distribution.

Japan, mainland China, Taiwan, Korea, Russia (southeast), India.

##### Host plant.

*Rubus buergeri* Miq., *R. trifidus* Thunb. (laboratory reared).

##### Remarks.

Our identification of this species is based on characters of external morphology (Motschulsky 1861; Inoue 1982; Li et al. 2012; Sasaki and Yamanaka 2013; Lu and Du 2020) and male genitalia (Li et al. 2012; Lu and Du 2020). The species status was confirmed by DNA barcodes.

#### Nagiella quadrimaculalis

Taxon classificationAnimaliaLepidopteraCrambidae

(Kollar & Redtenbacher, 1844)

F6509A54-AAB6-5E7D-A9AF-673568C41C33

Figures 2E, F , 5A–C , 8A–C

Scopula quadrimaculalis Kollar and Redtenbacher 1844: IV: 492.
Coptobasis quadrimaculalis : Lederer, 1863: 429–430; pl. 16 fig. 12.
Nagia desmialis Walker, 1866: 1320.
Sylepta quadrimaculalis : Shibuya, 1928: 229; pl. 8 fig. 14; Shibuya 1929: 189.
Nagia quadrimaculalis : Mutuura, 1957: 122, pl. 21 fig. 635.
Pleuroptya quadrimaculalis : Inoue, 1982: 1: 343; 2: 234, 454, pl. 40 fig. 17; Li et al. 2012: 624–625, pl. 18 fig. 415; Yamanaka 1995: 187, pl. 125 fig. 21; Sasaki and Yamanaka 2013: 81, 451.
Nagiella quadrimaculalis : Munroe, 1976: 878; Ullah et al. 2017: 70–72, figs. 2B, 4B, table 3; Lu and Du 2020: 149, fig. 6, figs. 11, 14.

##### Material examined.

Japan: 1♂, Marumori, Onikôbe, Narugo, Miyagi Pref., 30 Jul. 1997, M. Tanaka leg. (NSMT-I-L-75588); 1♂, Kirei-pass, Sumison, Miyazaki Pref., 9 Jul. 1992, Y. Yanagita leg. (NSMT-I-L-75592); 1♀, Hiromorigawa, Akita Pref., 14 Aug. 1988, A. Sasaki leg. (NSMT-I-L-75583); 1♀, Garo-Kyo, Fujisato Town, Akita Pref., 28 Jul. 2002, A. Sasaki leg. (NSMT-I-L-75584); 1♀, Yoroibata-Dam, Tazawako Town, Akita Pref., 26 Aug. 1989, A. Sasaki leg. (NSMT-I-L-75585); 2♂, Tose, Tamagawa, Tazawako Town, Akita Pref., 21 Aug. 1993, A. Sasaki leg. (NSMT-I-L-75586, 75587); 1♂, Futamata-Spa, Fukushima Pref., 6 Aug. 1967, T. Ebato leg. (NSMT-I-L-75462); 1♀, Houshi, Gunma Pref., 19 Jul. 1957, T. Ebato leg. (NSMT-I-L-75466); (NSMT-I-L-75472), 1♂, Kawaburu-Spa, Gunma Pref., 1 Jul. 1967, T. Ebato leg.; 1♂, Mt Takao, Tokyo To, 23 Jun. 1951, T. Haruta leg. (NSMT-I-L-75479); 1♀, ditto, 10 Jul. 1960, T. Ebato leg. (NSMT-I-L-75463); 1♂, ditto, 26 Jun. 1959, T. Ebato leg. (NSMT-I-L-75464); 1♀, ditto, 27 Jun. 1959, T. Ebato leg. (NSMT-I-L-75474); 1♂, Nippara, Tokyo To, 8 Aug. 1961, T. Ebato leg. (NSMT-I-L-75465); 1♀, same data as for preceding (NSMT-I-L-75477); 2♂, ditto, 2 Sep. 1961, T. Ebato leg. (NSMT-I-L-75475, 75476); 1♀, Mt Mitake, Tokyo To, 27 Aug. 1960, T. Maenami leg. (NSMT-I-L-75486); 1♂, ditto, 20 Jun. 1996, U. Jinbo leg. (NSMT-I-L-36059); 1♀, Ohnita, Ohme City, Tokyo To, 18 Aug. 1996, U. Jinbo leg. (NSMT-I-L-75634); 1♂, Yuzurihara, Yamanashi Pref., 1 Sep. 1945, T. Ebato leg. (NSMT-I-L-75471); 1♂, ditto, 2 Sep. 1945, T. Ebato leg. (NSMT-I-L-75469); 1♂, ditto, 5 Sep. 1945, T. Ebato leg. (NSMT-I-L-75470); 1♂, ditto, 23 Sep. 1954, T. Ebato leg. (NSMT-I-L-75468); 1♂, Sagashio-Spa, Yamanashi Pref., 9 Aug. 1969, T. Ebato leg. (NSMT-I-L-75473); 2♂, Ashiyasu, Yamanashi Pref., 16 Jul. 1977, T. Ebato leg. (NSMT-I-L-75480, 75482); 1♂, ditto, 6 Aug. 1977, T. Ebato leg. (NSMT-I-L-75483); 1♀, ditto, 19 Jul. 1980, T. Ebato leg. (NSMT-I-L-75478); 1♂, Nishiyama-Spa, Yamanashi Pref., 17 Aug. 1981, T. Ebato leg. (NSMT-I-L-75481); 1♀, Hirayu, Gifu Pref., 7 Aug. 1953, T. Haruta leg. (NSMT-I-L-75467); 1♂, Gujo-Rokunori, Gifu Pref., 1 Jul. 1966, S. Sawatani leg. (NSMT-I-L-75589); 1♂, Osugi-dani, Wakayama Pref., 4 Aug. 1976, S. Nakatani leg. (NSMT-I-L-75484); 1♂, Shimakawa-Osugi-dani, Wakayama Pref., 5 Jul. 1975, S. Nakatani leg. (NSMT-I-L-75485); 1♂, Tatsumi-tôge, Tottori City, Tottori Pref., 35.32°N, 134.01°E, 670 m, 2 Jul. 2019, Y. Matsui leg.; 2♀3♂, Mt Daisen, Kôfu Town, Tottori Pref., 35.35°N, 133.55°E, 910 m, 30 Sep.–29 Oct. 2017, and 18 Apr. 2018 (F_1_ emerged), Y. Matsui leg.; 1♂, Suemochi, Shikano Town, Tottori City, Tottori Pref., 35.45°N, 134.09°E, 220 m, 1 Jun. 2020, Y. Matsui leg.; 1♂, Kôchi, Shikano Town, Tottori City, Tottori Pref., 35.40°N, 134.00°E, 495 m, 12 Jun. 2020, Y. Matsui leg.; 2♀1♂, ditto, 12–16 Aug. 2020 (F_1_ emerged), Y. Matsui leg.; 1♂, Sourokubara, Tottori City, Tottori Pref., 35.46°N, 134.11°E, 110 m, 19 Jul. 2020 (larvae: collected from *Rubus buergeri*), 20 Aug. 2020 (emerged), Y. Matsui leg.; 1♀1♂, Toyofusa, Daisen Town, Tottori Pref., 35.41°N, 133.55°E, 680 m, 2 Sep. 2020 (larvae: collected from *R. palmatus*), 19–20 Oct. 2020 (emerged) Y. Matsui leg.; 1♂, Yakawa, Okuizumo Town, Shimane Pref., 35.10°N, 133.13°E, 680 m, 6 Sep. 2013, Y. Matsui leg.; 2♂, Omogokei, Kumakôgen Town, Ehime Pref., 33.72°N, 133.10°E, 700 m, 8 Jun. 2019, Y. Matsui leg.; 1♂, Shimomyo, Aira-Cho, Kagoshima Pref., 28 May 1992, Y. Yanagita leg. (NSMT-I-L-75590); 1♂, Tobi, Miyanojo-cho, Kagoshima Pref., 26 May 1992, Y. Yanagita leg. (NSMT-I-L-75591); 1♀, Mt Ishizukadake, I. Yakushima, Kagoshima Pref., 5–6 Aug. 1958, B.T. leg. (NSMT-I-L-75599); 1♂, same data as for preceding (NSMT-I-L-75600); 4♂, ditto, 17 Jul. 1970, K. Tobi leg. (NSMT-I-L-75601 to 75604); 1♀, same data as for preceding (NSMT-I-L-75605).

##### Diagnosis.

Adult (Fig. 2E, F). Forewing length 15.5–18 mm. This species is similar to *N. tristalis* and *N. inferior*, but can be distinguished by the following characters: forewing longer; vertex scales brown, darker than in *N. inferior*; cilia cream white at Cu_2_ to A_1+2_ for forewing, Cu_2_ to CuP for hindwing; subdiscal white spot of forewing quadrilateral or rounded, rather distinct; base of discal cell of hindwing partially white; gnathos of male genitalia slender and elongated (Fig. 5B); valva of male genitalia broader than in *N. tristalis* and *N. inferior*, with straight dorsal margin subapically (Fig. 5C); anterior apophysis of female genitalia slightly curved, expanded near bases, but not triangular (Fig. 8B); signum of female corpus brusae larger than that of *N. tristalis* and *N. inferior* (diameter 0.16–0.19 mm), with sharp projections at both edges of posterior margin (Fig. 8C). Ullah et al. (2017) provided diagnostic characters to distinguish this species from *N. occultalis*. Lu and Du (2020) mentioned genital differences between these species, as far as we can see, the specimen image of *N. bispina* by Lu and Du (2020) is externally distinguishable from this species by the cilia of each wing being concolorous with the ground color.

##### Distribution.

Japan, mainland China, Taiwan, Korea, Russia (southeast), Southeast Asia, Nepal, India.

**Figure 6.**
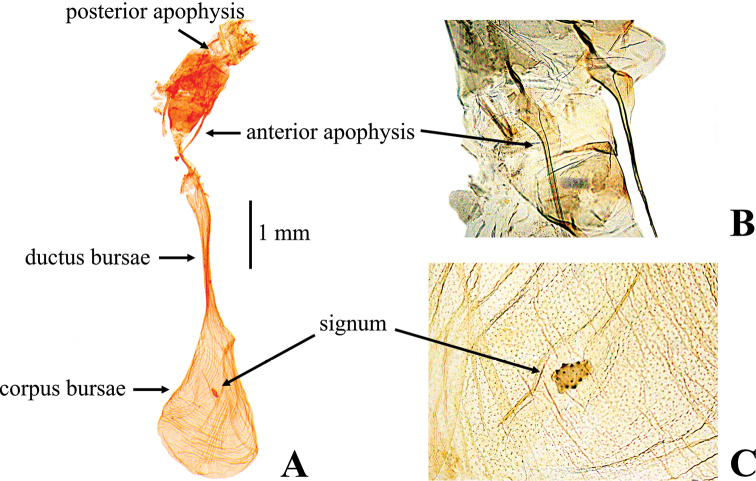
Female genitalia of *Nagiella tristalis* sp. nov. **A** whole genitalia **B** anterior apophysis, magnified view **C** signum, magnified view.

##### Host plant.

*Rubus buergeri* Miq., *R. palmatus* Thunb. (in the field), *R. buergeri*, *R. trifidus* Thunb. (laboratory reared).

##### Remarks.

*Nagia incomitata* Swinhoe, 1894 has long been considered a synonym of *N. quadrimaculalis*, but based on the investigation of the type specimen, Lu and Du (2020) considered it likely to belong to *Nosophora* Lederer, 1863. We also follow this taxonomic treatment.

**Figure 7.**
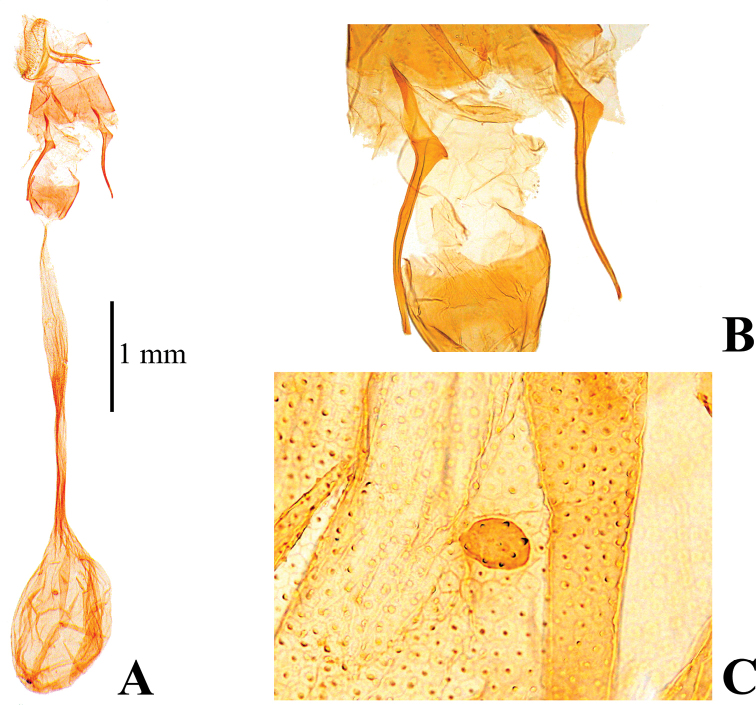
Female genitalia of *Nagiella inferior***A** whole genitalia **B** anterior apophysis, magnified view **C** signum, magnified view.

Our identification of this species in this study was based on external morphology (Kollar and Redtenbacher 1844; Inoue 1982; Li et al. 2012; Sasaki and Yamanaka 2013; Ullah et al. 2017; Lu and Du 2020) and male genitalia (Li et al. 2012; Ullah et al. 2017; Lu and Du 2020). The species status was confirmed by DNA barcodes.

**Figure 8.**
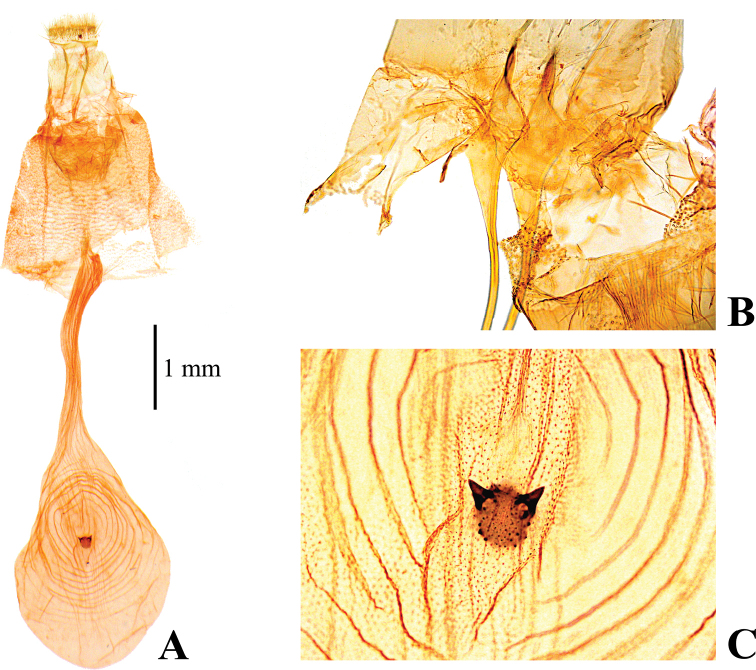
Female genitalia of *Nagiella quadrimaculalis***A** whole genitalia **B** anterior apophysis, magnified view **C** signum, magnified view.

## Discussion

Recently, the integration of DNA barcoding and morphological approaches has accelerated various stages of taxonomic studies, such as species identification and description, re-investigation of taxa, as well as detecting cryptic species, also in Spilomelinae (Sutrisno 2005; Haines and Rubinoff 2012; Mally et al. 2016; Ullah et al. 2017; Ullah et al. 2018; Lu et al. 2019; Mally et al. 2019; Yang et al. 2019; Lu and Du 2020). In the genus *Nagiella*, these approaches led to the discovery of *N. occultalis* and *N. bispina* from China (Ullah et al. 2017; Lu and Du 2020), as well as *N. tristalis* from Japan (this study). Combined morphological and molecular biological studies might lead to the discovery of additional new species of this genus also in other regions.

The NJ tree shows *N. inferior* + *N. tristalis* and *N. quadrimaculalis* + *N. occultalis* to be sister groups (Fig. 1). The morphological evidence also supports these relationships, i.e., the gnathos of the male genitalia is short and triangular in *N. inferior* and *N. tristalis* (Figs 3B, 4B), while that of *N. quadrimaculalis* and *N. occultalis* is elongated (Fig. 5B); the signum of the female corpus bursae lacks projections in *N. inferior* and *N. tristalis* (Figs 6C, 7C), while that of *N. quadrimaculalis* has projections (Fig. 8C), although that of *N. occultalis* is unknown.

As the species of the genus *Nagiella* are very similar in appearance to each other (except for *N. hortulatoides*), DNA barcoding (see Material and methods) may provide very useful information for the identification of species in this genus. However, the species information for this genus in BOLD probably contains some misidentifications. For example, BOLD:AAD8178 cluster contains a record named “*Pleuroptya inferior*”. This cluster can be identified as *N. quadrimaculalis* based on the results of this study and the specimen images in the deposited data. On the other hand, the sequences of *N. inferior* in this study corresponded to the BOLD:AAE4571 cluster, not BOLD:AAD8178. Therefore, “*Pleuroptya inferior*” in BOLD:AAD8178 is probably a misidentification. The user must judge whether the information in the database is based on correct identifications or not.

Host plant records of *Rubus buergeri* and *R. sieboldii* Blume for *N. inferior*, and *R. buergeri* for *N. quadrimaculalis* were known from Japan (Sasaki and Yamanaka 2013). However, in these published host plant records the *Nagiella* species may have been confused. In our laboratory, the three *Nagiella* species (including *N. tristalis*, reared from eggs) fed on *R. buergeri* and *R. trifidus*. In the field, we found larvae of *N. quadrimaculalis* feeding on *R. buergeri* and *R. palmatus*, and *N. tristalis* feeding on *R. buergeri.* In Tottori Prefecture, Japan, where the distribution of *N. tristalis* and *N. inferior* overlap, we could not find larvae of the latter species in the field, although where we found the larvae of the former species on *R. buergeri* in winter. This suggests that either 1) *R. buergeri* is not the native host plant of *N. inferior*, or that 2) *N. inferior* has a different overwintering strategy than *N. tristalis*. Contrary to our results, Fan and Piao (2013) recorded *Rhus chinensis* Mill. (Anacardiaceae) as a host plant for *N. quadrimaculalis*. The native host plants of the genus *Nagiella* needs further investigation.

The species of *Nagiella* have been placed in various genera, namely *Coptobasis* Lederer, *Pleuroptya* Meyrick, and *Sylepta* Hübner (e.g., Lederer 1863; Hampson 1899; Shibuya 1928; Inoue 1982; Irungbam et al. 2016). Munroe (1976) separated *Nagiella* from *Pleuroptya* and its related genera by the following characters: uncus short and wide, valva with a large oblique clasper, saccus relatively small and simple, and type of wing maculation (consisting of greyish ground color and a conspicuous white spot on each wing). Inoue (1982) placed *N. inferior* and *N. quadrimaculalis* in *Pleuroptya*, but no evidence for this treatment was provided. Inoue’s opinion was followed by many authors (e.g., Li et al. 2012; Sasaki and Yamanaka 2013; Irungbam et al. 2016). Kirti and Gill (2007) treated *Pleuroptya* as a synonym for *Patania* Moore, because the male genitalia of their respective type species share congeneric characters as follows: valva leaf-like, uncus with a truncate posterior margin, gnathos absent, tuba analis elongate, and cornutus present in phallus. Ullah et al. (2017) regarded *Nagiella* as a valid genus based on the following characters: gnathos present, valva broader than that of *Patania* with stout subapical setae, and phallus without cornutus. However, several characters such as the presence or absence of a gnathos and the presence or absence of a cornutus in the phallus are shared with some *Patania* species. For example, *P. clava* Xu & Du, 2016 possesses a developed, finger-like gnathos (Xu and Du 2016), *P. balteata* (Fabricius, 1798) has an elongated gnathos (Leraut 2005; Slamka 2013), *P. accipitralis* (Walker, 1866) is missing cornuti in the phallus (Leraut 2005), and *P. obfuscalis* (Yamanaka, 1998) possesses a bunch of setae medially on the costa of the valva (Yamanaka 1998). In addition, the male genitalia of *N. bispina* described by Lu and Du (2020) lack the gnathos and possess a hook-shaped cornutus. Therefore, we tentatively regard *Nagiella* as a valid genus based on the following available characters: large oblique clasper, wing maculation, and host plants. Although in *N. hortulatoides* the wing maculation is different, this taxon is obviously included in *Nagiella* based on characters of the male genitalia as shown by the phylogenetic results of Lu and Du (2020). Although the separation of *Nagiella* has been accepted by many authors, such as Kirti and Sodhi (2001), Rose (2002), Mally et al. (2019), and Nuss et al. (2003–2020), further comprehensive genitalic studies and also molecular phylogenetic analyses are indispensable to reveal details of the taxonomic status of *Patania*, *Pleuroptya*, and *Nagiella*.

## Supplementary Material

XML Treatment for Nagiella tristalis

XML Treatment for Nagiella inferior

XML Treatment for Nagiella quadrimaculalis
